# Some Observations on the Treatment of the Narrow and Irritable Stricture of the Urethra

**Published:** 1861

**Authors:** D. D. Slade

**Affiliations:** One of the Surgeons of the Boston Dispensary


### SOME OBSERVATIONS

ON THE TREATMENT OF THE

### NARROW AND IRRITABLE STRICTURE OF THE URETHRA.

By D. D. Slade, M. D., one of the Surgeons of the Boston Dispensary.

I propose to offer to the Society some practical observations upon the introduction of instruments in cases of narrow and irritable stricture of the urethra, more particularly for the purpose of relieving retention of urine. Much as has been written and spoken upon this subject, it is one, the important practical bearing of which will admit of its being frequently brought before us; for in our community especially, every medical man is liable to be called upon, at a moment's notice, to afford relief in the crisis of retention. If I was asked what common operation in surgery required the most tact, careful manipulation, and, above all, gentleness and patience, I should unhesitatingly say catheterism. Not but that any man may succeed, with more or less adroitness, in introducing a catheter into the bladder, provided the parts are in a perfectly normal condition; but let him meet with any obstruction, then his attempts may be completely foiled, unless by experience and constant practice he shall be prepared to overcome them.

It is a well-established rule at the present day, or at least it ought to be, that puncture of the bladder is never necessary, the cases of failure to arrive at the bladder through the natural passage, by well-directed and skillful manipulations, being so extremely rare. To be sure, puncture of the bladder is an operation which is constantly performed in Hospitals and elsewhere, but had these very cases fallen into skilful hands at the commencement of the retention, or had more patience and perseverance been practiced by the hospital surgeons themselves, no such extremity would have been resorted to, excepting under extremely rare circumstances. Prof. Syme, of Edinburgh, long ago publicly taught that there are *no* strictures capable of allowing the passage of urine, even in drops, which cannot be permeated by skilfully-directed efforts. Civiale, in his admirable cliniques, and in his works, assures us that puncture of the bladder is never necessary. Such, in fact, is the opinion of the best surgical authority at the present day.

In my own practice, I have been called to several cases of retention of urine where the method of treatment of which I

am about to speak, pursued with gentleness and perseverance, alone saved the patients from having the bladder punctured, that operation, in one case at least, having been determined upon by the attending physician.

Let us suppose, then, that we are called to treat a case of narrow, irritable stricture, where retention of urine has not actually taken place, but where, in fear of such a result, attempts have been made to pass the catheter without success. In such a case, we must have recourse at once to general treatment. Rest in bed, warm baths, laxatives, strict attention to diet, opiate enemata, and, above all, care not to introduce any instrument into the urethra, will be found soon to have their marked beneficial effects; the immediate tendency to retention will disappear, and by following up this plan of treatment for a sufficient length of time, we shall place the organs in the best possible condition for undergoing the proper local treatment.

On the other hand, let us suppose that we are called upon after retention of urine has occurred, and where immediate relief must be given, and where attempts to reach the bladder may or may not have been made. In such a case, the passage through the stricture must necessarily be extremely small, and therefore in order to pass an instrument through it, we must select one of a corresponding size. For this purpose, I always use one of these delicate gum-elastic bougies, some of which, as you see, are scarcely larger than an ordinary knitting-needle.

I prefer that the patient should be in bed, that he should be warmly covered, and that he should be particularly protected against any sudden chill. A bougie is then to be selected, of a size corresponding to the size of the stream passed, as nearly as may be, or to the presumed diameter of the constricted passage; this is to be carefully lubricated with lard, cold cream, cerate, or some other equally tenacious substance, which is greatly to be preferred to the olive oil so commonly in use. Thus prepared, the instrument is to be carried carefully down to the seat of the stricture, and, if possible, pushed on into it, the entrance of its extremity being at once known by the peculiar manner in which it is grasped. After a few moments' delay, the bougie, in the great majority of cases, may be pushed on into the bladder. This, however, it must be borne in mind, is not always necessary; the mere presence of the instrument at the seat of the obstruction is generally sufficient to overcome the spasmodic action upon which the retention depends. The only difficulty in carrying these delicate instruments down to the stricture, is from their becoming entangled in the various lacunæ, which, as is well known, are greatly enlarged in this disease. This difficulty, however, can be obviated by making traction upon the penis, so as to put the mucous membrane upon a stretch—or, in those cases which will admit of it, making use of the probe-pointed or olive-shaped bougie, of which I shall speak.

Where one or more false passages exist, by certain careful rotatory movements given to the instrument, we shall succeed in engaging the point within the stricture more speedily and safely with these delicate bougies, than by any other means. For this very purpose, M. Leroy made use of gum-elastic bougies which were bent in the form of a cork-screw, and which he often found extremely useful. Whatever form of instrument may be selected, I cannot too strongly enforce the necessity of using the greatest gentleness in its introduction. Anything like violence or even roughness, will not only give our patient great and unnecessary pain, but will be sure to be followed by an increased spasmodic action of the parts, which will defeat all our efforts. M. Civiale never could say too much on this point, which certainly is the basis of all success in catheterism.

Mr. Henry Thompson, of London, has recently suggested a method of protecting the mucous membrane from injury, and of rendering the introduction of small instruments more easy, particularly in these very cases of narrow stricture, which, on trial, will be found very useful. It consists in the simple method of applying the oil to the urethra itself, and very freely, rather than to the instrument. In order to effect this, he says, the nozzle of a common glass syringe, containing from four to six drachms of pure olive oil, should be introduced into the urethra as far as it will go, the external meatus being at the same time closed upon the nozzle by the forefinger and thumb of the left hand, so that none can escape. Gentle pressure being now made upon the piston-rod, the oil gradually finds its way down to the stricture; and if this be very narrow, the urethra in front of it slowly fills and becomes slightly distended; but as the piston continues to descend, the oil will gradually pass through the stricture and onward into the bladder, thoroughly lubricating every part of the canal. At the moment the oil passes through the stricture, the operator may sometimes distinctly perceive a slight, but very complete sensation communicated to the hand, of resistance overcome, and partial collapse of the previously distended urethra in front. The syringe is then to be removed, the finger and thumb still commanding the meatus of the urethra so that no oil escapes. The smallest catheter may now be introduced, and made to traverse the urethra—at all events as far as the stricture—with very little or none of that difficulty arising from the catching of its point against the walls of the passage, so often experienced with very small instruments, and which renders so much care necessary in their employment. But what is more, when arrived at the stricture, the instrument, if adapted in size, will gradually pass through it; or, at least, the probability of its doing so is greatly increased. The narrowed channel has not only been thoroughly lubricated, but somewhat distended by the mechanical pressure of the column of oil which has passed through it; and this sometimes occurs to an extent which affords no inconsiderable amount of aid to the operator. Patients suffering from very irritable stricture have experienced so much less pain from the passage of a catheter after the injection of oil, that I have been repeatedly requested by them to employ it on subsequent occasions.

I alluded to the probe-pointed bougie as being extremely useful in many cases of stricture. The delicate extremity of the bougie being armed with this olive-shaped button, prevents it from being caught in the lacunæ as it is passed down. So, also, under certain circumstances, it will be found that this form of bougie can be more readily insinuated into and even passed through one of these narrow strictures than any other. By means of this, also, we can easily pass down ointments of various kinds.

Mr. Thompson has recently advocated also the use of a probe-pointed catheter. This instrument resembles in form, length and curve the ordinary catheter, and is made of silver. For the last two inches, however, it is perfectly solid, the extremity being in fact, a delicate metal probe. However small it may be necessary to have the instrument, so small can this probe-pointed extremity be made. The hollow part of the instrument commences at about two and a half inches from the point, and a small eye is placed on the inner aspect of the curve. From this part the instrument gradually increases in diameter. The whole is strengthened by a small steel rod or stylet, which accurately fills the interior, and to which the handle is affixed. The small eye can thus be kept clear of mucus and other matters. Mr. Thompson says: "when the stricture has been passed, considerable care is necessary in guiding onward the point through the canal behind, to prevent it becoming engaged in the enlarged lacunæ, which are commonly found in the dilated urethra behind an old stricture. This being safely accomplished, and the stylet removed, the urine will issue by drops only, on account of the small size of the eye, but nevertheless in a manner which will soon relieve the patient, and which at once assures the surgeon of his complete success.

I cannot myself see any particular advantage to be derived from such an instrument as the one just described. After passing through the stricture, a considerable portion of the instrument must be pushed on into the bladder, beyond the seat of the difficulty, before any urine could pass through the eye, and that too without any certainty that irreparable mischief may not be done to the parts. The probe-pointed bougie seems to me to be a much safer instrument, and much better adapted, in the majority of cases, to the proper treatment of narrow stricture. After either form of bougie of which I have spoken has been passed, and the retention, if it exists, has been relieved, their use can be followed by larger instruments of the same material or the metallic ones may be substituted.

We may not always succeed in passing instruments of such tenuity at the first trial, but by affording the parts an opportunity to rest, and the spasmodic action to subside, especially in those cases where violent measures have been pursued, success will finally reward our efforts. The perfect relaxation of all spasmodic action under the use of anæsthetic agents, often renders their administration extremely useful in our treatment of retention from a contracted stricture. I am of opinion that this is not borne in mind so generally as it ought to be.

Temporary dilatation is, without doubt, the safest and surest method of treating organic stricture. Although slow, at the same time it can be easily managed and can be suspended at any moment, according as circumstances require, and, above all, does not prevent the patient from pursuing his usual avocation—and for the early treatment of narrow, irritable stricture, the use of gum-elastic or wax bougie is far preferable to metallic instruments. I have seen patients who have suffered so much from the passage of small metallic instruments, that they have not been willing to allow their farther use, but have made rapid progress under the employment of flexible instruments. When, however, the dilation has proceeded so far that a No. 5 or 6 bougie passes with ease, then these may be laid aside and metallic instruments substituted.

I cannot close my remarks better than by quoting the words of Mr. Solly. "There is another thing to be remembered in the treatment of stricture; never be ashamed to leave the bedside of a patient without succeeding in passing a bougie. I am told that a hospital surgeon, now deceased passed a sleepless night from vexation, if he failed to introduce an instrument into the bladder in presence of his pupils. Such a man must have made many a false passage. Every good surgeon will fail occasionally in the introduction of a bougie, but no good surgeon ought to make a false passage, though a skilful surgeon will sometimes do it, when his temper or his pride rules his hand, instead of his reason and his conscience."

*Boston Journal.*

